# Phytoliths Analysis for the Discrimination of Foxtail Millet (*Setaria italica*) and Common Millet (*Panicum miliaceum*)

**DOI:** 10.1371/journal.pone.0004448

**Published:** 2009-02-12

**Authors:** Houyuan Lu, Jianping Zhang, Naiqin Wu, Kam-biu Liu, Deke Xu, Quan Li

**Affiliations:** 1 Institute of Geology and Geophysics, Chinese Academy of Sciences, Beijing, China; 2 Department of Oceanography and Coastal Sciences, Louisiana State University, Baton Rouge, Louisiana, United States of America; Ecole Normale Superieure, France

## Abstract

Foxtail millet (*Setaria italica*) and Common millet (*Panicum miliaceum*) are the oldest domesticated dry farming crops in Eurasia. Identifying these two millets in the archaeobotanical remains are still problematic, especially because the millet grains preserve only when charred. Phytoliths analysis provides a viable method for identifying this important crop. However, to date, the identification of millet phytoliths has been questionable, because very little study has been done on their morphometry and taxonomy. Particularly, no clear diagnostic feature has been used to distinguish between Foxtail millet and Common millet. Here we examined the anatomy and silicon structure patterns in the glumes, lemmas, and paleas from the inflorescence bracts in 27 modern plants of Foxtail millet, Common millet, and closely related grasses, using light microscopy with phase-contrast and microscopic interferometer. Our research shows that five key diagnostic characteristics in phytolith morphology can be used to distinguish Foxtail millet from Common millet based on the presence of cross-shaped type, regularly arranged papillae, Ω-undulated type, endings structures of epidermal long cell, and surface ridgy line sculpture in the former species. We have established identification criteria that, when used together, give the only reliable way of distinguishing between Foxtail millet and Common millet species based on their phytoliths characteristics, thus making a methodological contribution to phytolith research. Our findings also have important implications in the fields of plant taxonomy, agricultural archaeology, and the culture history of ancient civilizations.

## Introduction

Phytoliths are microscopic silica bodies that precipitate in or between cells of living plant tissues, and are widespread in all types of plants and all their different organs and structures, from roots to culms to inflorescences [1]–[4]. They are especially abundant, diverse, and distinctive in the grass family (Poaceae). Phytoliths are released from plant tissues when they are decayed, burned, or digested. Released phytoliths thus become microfossils of the plants that produce them. The development and application of phytolith techniques in archaeology have led to major advances in investigating plant use and subsistence patterns in regions where preservation of macrobotanical remains is poor [2], [3], [5]–[13].

Foxtail millet (*Setaria italica*) and Common millet (also known as Broomcorn millet, *Panicum miliaceum*), belong to Panicoideae of Poaceae, are considered to be dry farming cereals that form the oldest cultivated foods known to humans [14]–[16]. They were staple foods in the Far East (China, Japan, Russia, and India) and even in the entire Eurasian continent prior to the popularity of rice and wheat, and are still important foods in the semi-arid regions today [14], [17], [18]. However, the archaeobotanical remains of Foxtail millet and Common millet are difficult to distinguish mainly due to the very small sizes of these grain crops – often less than 2∼3 mm in length, and their very similar shapes [8], [19], [20]. A previous study has also considered at some length how the charred de-husked grains of various native millet species might have been systematically misidentified [21]. Moreover, the identification of millet phytoliths has also been questionable [8], [20], [22], because very little study has been conducted on millet phytoliths, thus no clear diagnostic feature has been used to distinguish Foxtail millet from Common millet [8].

The inflorescence bracts in Poaceae are characterized by phytoliths with specific morphological characteristics, hence their taxonomic significance [23]. Wynn Parry and Smithson (1966) used light microscopy to record silicification of the various epidermal cell types and cuticle of the inflorescence bracts from various British grass genera [24]. Subsequently, a series of such studies has emerged utilizing the newer techniques of scanning electron microscopy (SEM) focusing on cereals and grasses, including descriptions for barley, oats, rice, rye and species of *Panicum*, *Echinochloa* and *Dicanthelium* [23], [25], [26]. Pearsall et al. (1995) and Zhao et al. (1998) used morphometric analysis to distinguish between rice and wild *Oryza* phytoliths [6], [12]. Tubb et al (1993) and Ball et al. (1999) developed paradigms using morphometric analysis for distinguishing between inflorescence phytoliths produced by several species of wheat and barley [27], [28]. Berlin et al. (2003) used these morphometric paradigms to identify *Triticum aestivum* in residues taken from ceramics at Tel Kedesh, Israel [29]. This paper reports the first attempt to determine if phytoliths analysis of the inflorescences bracts can be an effective tool for discriminating between Foxtail millet (*S. italica*) and Common millet (*P. miliaceum*).

## Materials and Methods

We examined modern phytoliths from twenty-seven species of domesticated and wild Paniceae. Domesticated species include nine species of *S. italica* L. Beauv. and twelve species of *P. miliaceum* L.; wild species include two species of *P. bisulcatum* Thunb., *S. plicata* (Lam.) T. Cooke, *S. glauca* (Linn.) Beauv., *S. viridis* (L.) Beauv., and *Echinochloa crusgalli* (L.) Beauv. Of these 27, four species were sampled from annotated folders at the National Crop Gene Bank of China, Chinese Academy of Agricultural Sciences (CAAS); six species were sampled from the Culture Museum of Cishan, Wuan, Hebei Province, China; fourteen species were sampled from Institute of Geology and Geophysics, Chinese Academy of Sciences, Beijing, China; and three species were sampled from East China Normal University, Shanghai, China. The folders contained samples of field collections by many investigators. For passport data on the plants, see Table 1.

**Table 1:** Passport information on the plants studied.

Source	No.	Species	Breed name	Province	Locality Information
NCGC	S1	*Setaria italica* (L.) Beauv.	Ai hui dang di zhong	Hei longjiang	
NCGC	S2	*Setaria italica* (L.) Beauv.	Fa gu 130-80		
NCGC	S3	*Setaria italica* (L.) Beauv.	Fa gu 45-81		
NCGC	P1	*Panicum miliaceum* L	64 shu 120		
CMCS	S4	*Setaria italica* (L.) Beauv.	Zhu xieqing	Hebei	
CMCS	S5	*Setaria italica* (L.) Beauv.	Dong huigu	Hebei	
CMCS	S6	*Setaria italica* (L.) Beauv.	Cixuan 6	Hebei	
CMCS	S7	*Setaria italica* (L.) Beauv.	Jigu 14	Hebei	
CMCS	S8	*Setaria italica* (L.) Beauv.	Cishan dang di gu	Hebei	36.57°N, 114.111°E, Altitude 264 m
CMCS	P2	*Panicum miliaceum* L	Cishan dang di shu	Hebei	36.57°N, 114.11°E, Altitude 270 m
IGG	S9	*Setaria italica* (L.) Beauv.	Jiaxiang dang di gu	Shandong	35.483°N, 116.192°E, Altitude 40 m
IGG	P3	*Panicum miliaceum* L	Xifeng dang di meizi	Shanxi	35.766°N,107.683°E, Altitude 1283 m
IGG	P4	*Panicum miliaceum* L	Xifeng dang di meizi	Shanxi	35.766°N, 107.683°E, Altitude 1273 m
IGG	P5	*Panicum miliaceum* L	Xifeng dang di meizi	Shanxi	35.766°N, 107.683°E, Altitude 1260 m
IGG	P6	*Panicum miliaceum* L	Jinzhong dang di shu	Shanxi	37.664°N, 112.722°E, Altitude 790 m
IGG	P7	*Panicum miliaceum* L	Jinzhong dang di shu	Shanxi	37.664°N, 112.722°E, Altitude 790 m
IGG	P8	*Panicum miliaceum* L	Jiaxiang dang di shu	Shandong	35.483°N, 116.192°E, Altitude 40 m
IGG	P9	*Panicum miliaceum* L	Qinan dang di meizi	Gansu	34.984°N, 105.533°E, Altitude 1442 m
IGG	P10	*Panicum miliaceum L*	Qinan dang di meizi	Gansu	34.984°N, 105.533°E, Altitude 1430 m
IGG	P11	*Panicum miliaceum L*	Licheng dang di shu	Shanxi	36.482°N, 113.396°E, Altitude 772 m
IGG	P12	*Panicum miliaceum L*	Licheng dang di shu	Shanxi	36.482°N, 113.392°E, Altitude 770 m
ECNU	SP1	*Setaria plicata* (Lam.) T. Cooke	Zhouye gouweicao	Fujian	
ECNU	SG1	*Setaria glauca* (Linn.) Beauv.	Jin gouweicao	Anhwei	
ECNU	PB1	*Panicum bisulcatum* Thunb.	Kang ji	Anhwei	
IGG	SV1	*Setaria viridis* (L.) Beauv	Qing gouweicao	Beijing	40.069°N, 116.441°E, Altitude 30 m
IGG	PB2	*Panicum bisulcatum* Thunb.	Kang ji	Beijing	40.070°N, 116.440°E, Altitude 33 m
IGG	E1	*Echinochloa crusgalli* (L.) Beauv	Bai cao	Beijing	40.069°N, 116.440°E, Altitude 31 m

Notes: NCGC is National Crop Gene bank of China, Chinese Academy of Agricultural Sciences (CAAS). CMCS is Culture Museum of Cishan, Wuan, Hebei Province, China. IGG is Institute of Geology and Geophysics, Chinese Academy of Sciences, Beijing, China. ECNU is East China Normal University, Shanghai, China.

**Figure 1:**
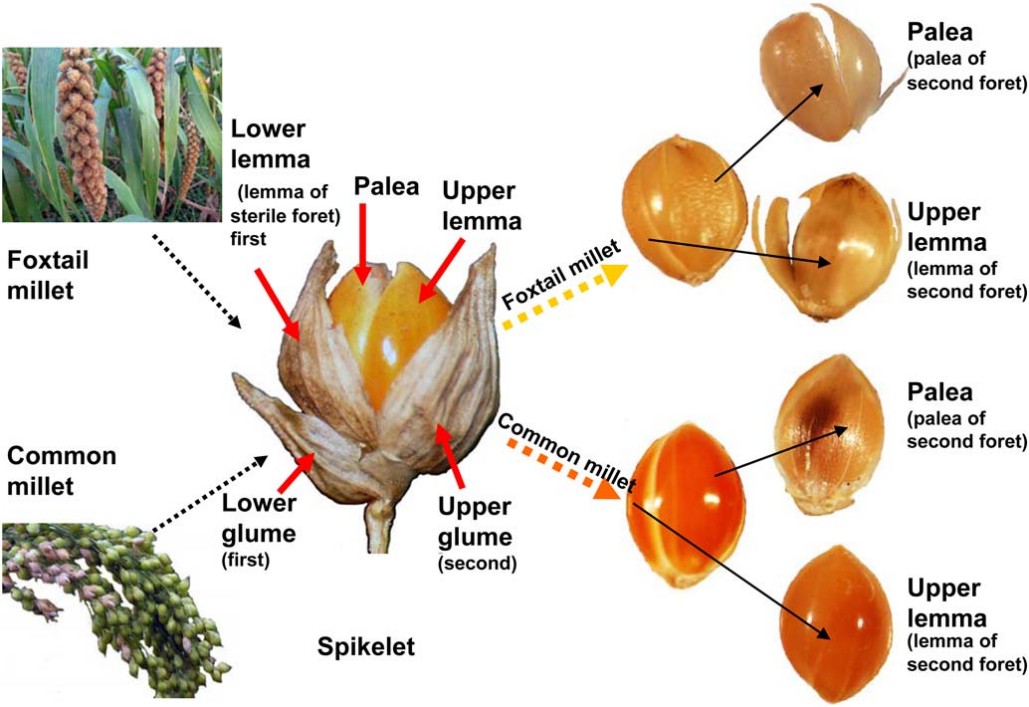
Illustrations of spikelet and grain of millets with botanical terms.

In this study, we dissected the spikelet of modern plants into five parts, including lower glume, upper glume, lower lemma (lemma of sterile floret), upper lemma, and palea [30] (see Figure 1) for phytolith analysis. Palea can be divided into “palea of first floret” and “palea of second floret”. However, in both genera *Setaria* and *Panicum*, the palea of first floret is atrophied to a very small and membranous organ and sometimes becomes lost in the spikelet. Thus, in this study, we used “palea” for the “palea of second floret”. The five parts of spikelet were prepared in the following manner.

(i) Each part of spikelet was cleaned with distilled water in a water bath to remove adhering particles. (ii) All samples were placed in 20 ml of saturated nitric acid for over 12 h to oxidize organic materials completely. (iii) The solutions were centrifuged at 2000 r.p.m. for 10 min, decanted and rinsed twice with distilled water, and then rinsed with 95% ethanol until the supernatants were clear. (iv) The phytolith sediments were transferred to storage vials. The residual subsamples were mounted onto microscopic slides in Canada Balsam medium for photomicrography and in liquid medium for counting, measuring, and line drawing. (v) Light photomicrography (phase-contrast, and microscopic interferometer) at 400× magnification was used to determine their anatomy and silicon structure patterns in the glumes, lemmas, and paleas. (vi) Phytolith parameters were measured using computer-assisted image analysis.

## Results

### Phytolith morphology of the lower lemma and glumes

Micromorphological characters of the lower lemma and glumes are generally similar for each millet sample based on our observation and statistic of all samples. Silicification always occurs in the short cells (silica cells), and occasionally occur in some of the long cells, micro-hairs, macro-hairs, and stomata in the lower lemma and glumes of both Foxtail millet and Common millet.

**Figure 2:**
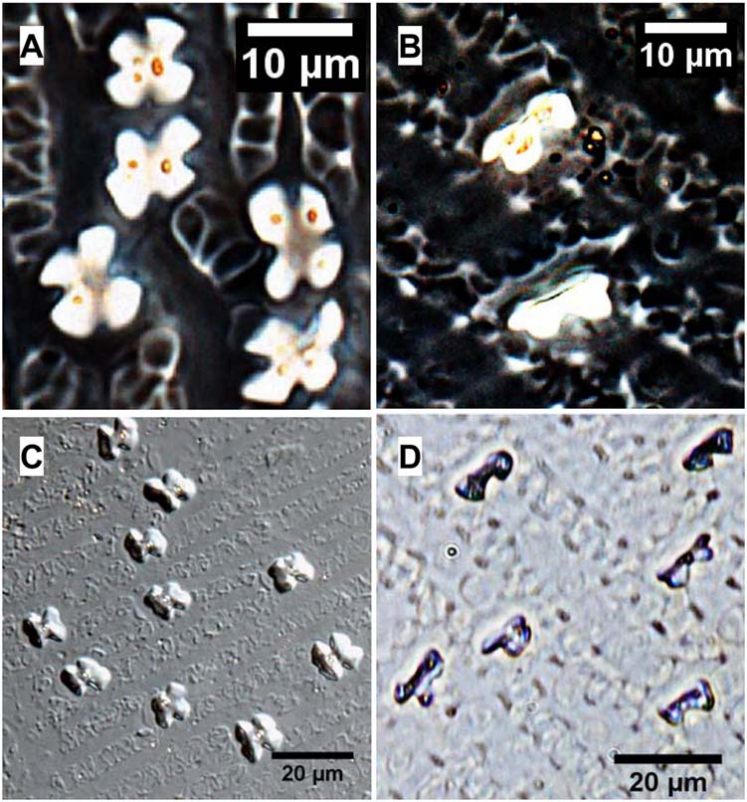
Comparison of phytolith morphology in the lower lemma and glume for Foxtail millet and Common millet. (A), (C) Cross-shaped type of phytoliths from *S. italica*; (B), (D) Bilobe-shaped type of phytoliths from *P. miliaceum*.

The shape of the silica bodies formed in the short cells of Foxtail millet is different from Common millet (Figure 2). Cross-shaped (ratio length∶ width≈1∶1) phytoliths are found in Foxtail millet, and tend to increase in size variation (range 4.46–9.98 µm; average 7.55±1.17 µm, N = 208) toward the central part of the lower lemma and glumes. However, the Common millet has bilobe-shaped (dumbbell-shaped, ratio length∶ width≈1∶2) phytoliths with the two endings distinctly branched. The bilobe-shaped phytoliths are oriented with the bar cross at a right angle to the long cells (Figure 2D), and tend to increase in size (length) variation (range 8.08–15.05 µm; average 10.87±1.43 µm, N = 198) toward the central part of the lower lemma and glumes.

Other silicified cells, including long cells, micro-hairs, macro-hairs, and stomata, are without any characteristic shape, and not easily identified in phytoliths. This suggests that the cross-shaped type is formed in the lower lemma and glumes of *S. italica*, and the bilobe-shaped type is formed in those of *P. miliaceum*.

### Phytolith morphology of the upper lemma and palea

#### Phytolith morphology of the upper lemma

**Figure 3:**
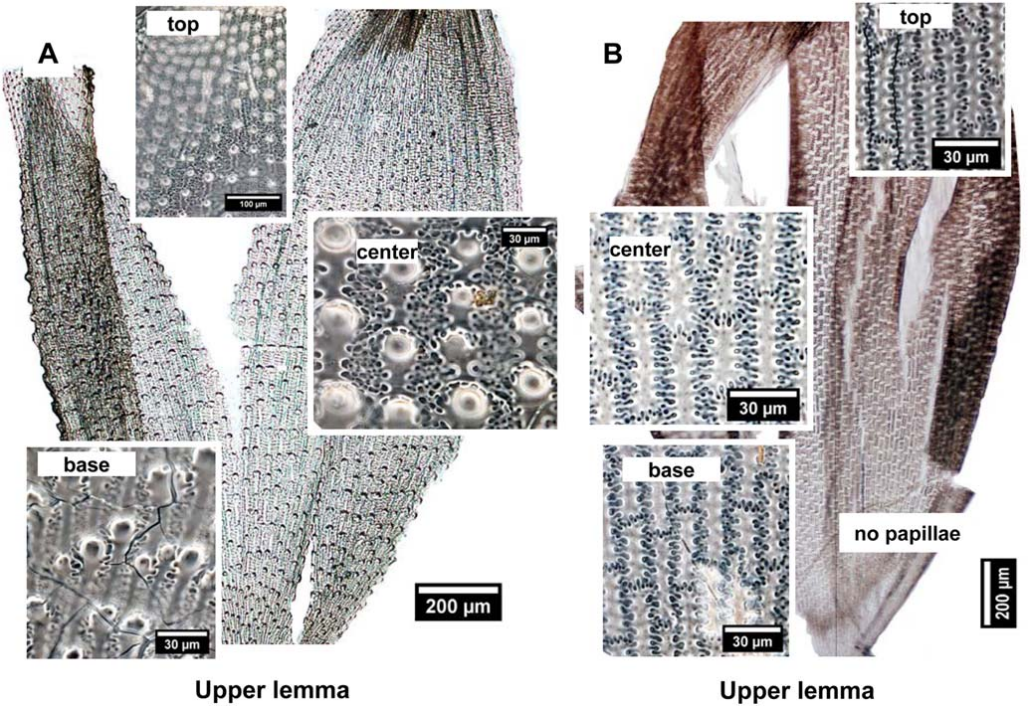
Comparison of the characteristics of deposited silicon in the surface of the upper lemma for the two millet species. (A) Foxtail millet upper lemma produces papillae. (B) Common millet upper lemma does not produce papillae.

**Figure 4:**
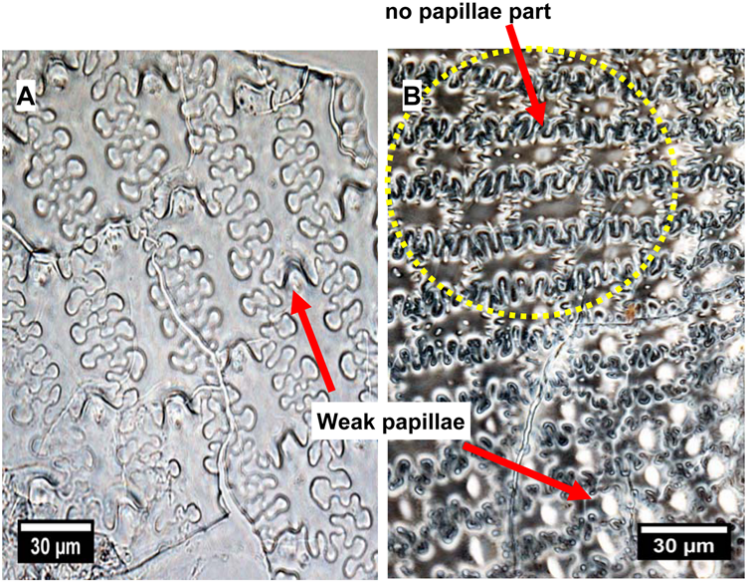
Papillae distribution on surfaces of the upper lemma from Foxtail millet. (A) Weak papillae formed on surfaces of the upper lemma are peculiar to some *S. italica*. (B) No papillae area also formed on surfaces of the upper lemma from some *S. italica*.

The presence and form of papillae visible under microscopy on the upper lemmas are important characteristics for identifying *S. italica* (Figure 3). Upper lemmas of *S. italica* have distinct papillae by the silicification of the surface, cell wall, and/or lumen of epidermal papillae cells. The bases of papillae are typically suborbicular with semicircular to sinuous to irregular margins. They typically have a single papillate and tend to decrease in size variation (papillae diameter ranges between 5 µm and 30 µm) from center toward the base and top part of the upper lemma (Figure 3A), but may also be scutiform or dome-shaped, and lacking a clear projection weak papillae. No papillae area is formed on the surfaces of the upper lemma of some *S. italica*. (Figure 4).

The upper lemma of *P. miliaceum* is characterized by a smooth surface without any papillae (Figure 3B) in every area of all samples. Therefore, the papillae formed on surfaces of the upper lemma are peculiar to *S. italica*.

#### Phytolith morphology of the palea

**Figure 5:**
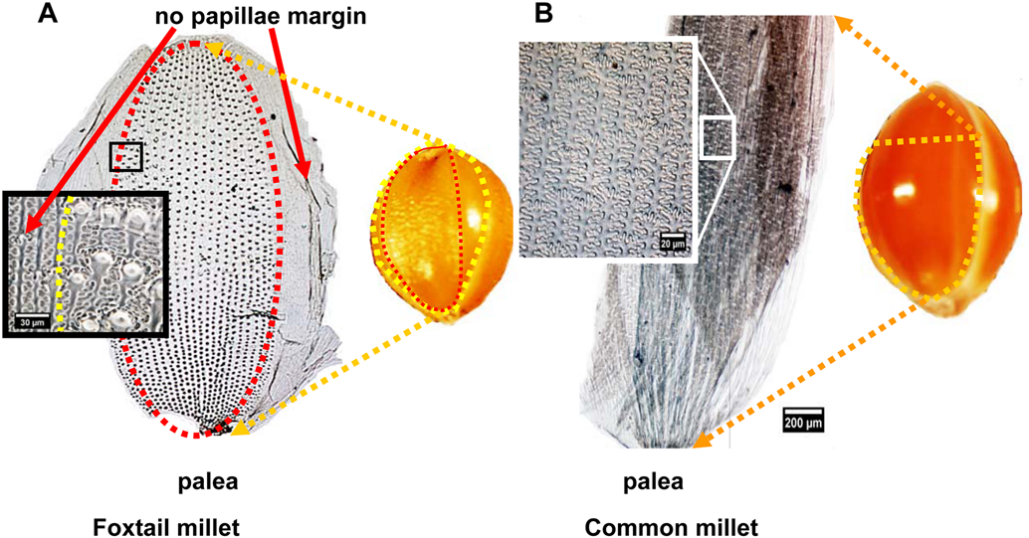
Comparison of the characteristics of deposited silicon in the surface of the palea for the two millet species. (A) Regularly arranged papillae formed on center surfaces of the palea are peculiar to *S. italica*. (B) Surfaces of the palea of Common millet do not produce papillae.

Regularly arranged papillae are consistently found in center surfaces of the palea of *S. italica*, and tend to decrease in size variation (papillae diameter ranges between 5 µm and 25 µm) toward the base and top of the palea (Figure 5A). *P. miliaceum* does not have any papillae in the entire area of palea in all samples (Figure 5B).

The phytolith morphology of *S. italica* and *P. miliaceum* can be clearly distinguishable based on the presence or absence of papillae. Regularly arranged papillae on the surface of the upper lemma and palea are peculiar to *S. italica*. However, it should be cautioned that the identification of *P. miliaceum* cannot be confirmed based solely on the absence of papillae, because papillaes may sometimes vanish into a smooth surface on the surface of upper lemma and palea in *S. italica*.

#### The undulated patterns of epidermal long cell in the upper lemma and palea

**Figure 6:**
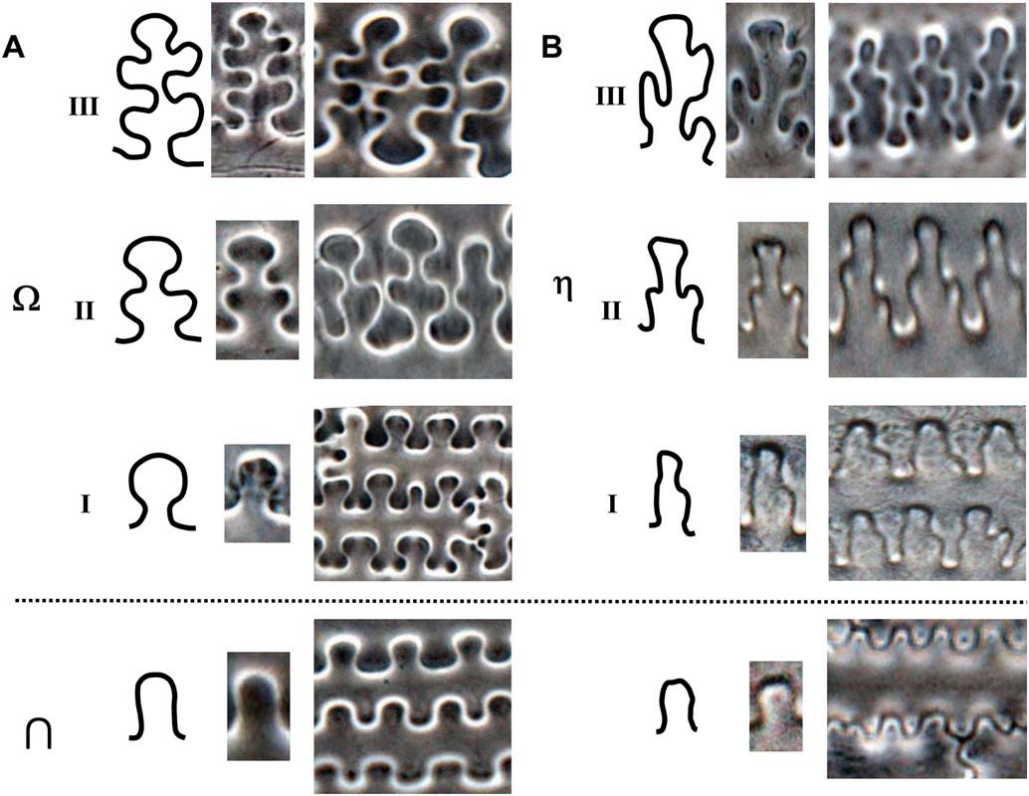
Comparison of the undulated patterns of epidermal long cells in the upper lemma and palea for two the millet species. (A) The epidermal long cell walls are Ω-undulated in *S. italica*, and (B) η-undulated in *P. miliaceum*.

By means of light microscopy with phase-contrast and microscopic interferometer the surface undulated patterns of epidermal long cell walls in the upper lemma and palea from both *S. italica* and *P. miliaceum* can be divided into two distinctly different types by means of particularity analysis (Figure 6). The epidermal long cell walls are Ω-undulated (undulations rounded, wider toward the apex and narrower at the base) in *S. italica*, and are η-undulated in *P. miliaceum*. Ω-undulated types can produce branching subordinate Ω subtypes based on the degree of undulations as Ω I, II, III. Similarly, η-undulated types can also produce branching subordinate η-undulated subtypes, including ηI, II, III (Figure 6).

It is noteworthy that there are also ∩-undulated types (Figure 6), which only occur at the narrow margin part of the lemmas and palea. It is very difficulty or impossible to distinguish them, because their very simple and similar morphology occurs in both Foxtail millet and Common millet.

**Figure 7:**
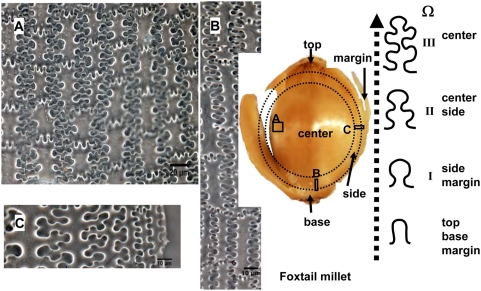
Undulated patterns transformation of epidermal long cell walls in the upper lemma and palea of Foxtail millet. (A), (B), and (C) showing the different designs of phytoliths at center, base, and side of lemma of Foxtail millet, respectively.

**Figure 8:**
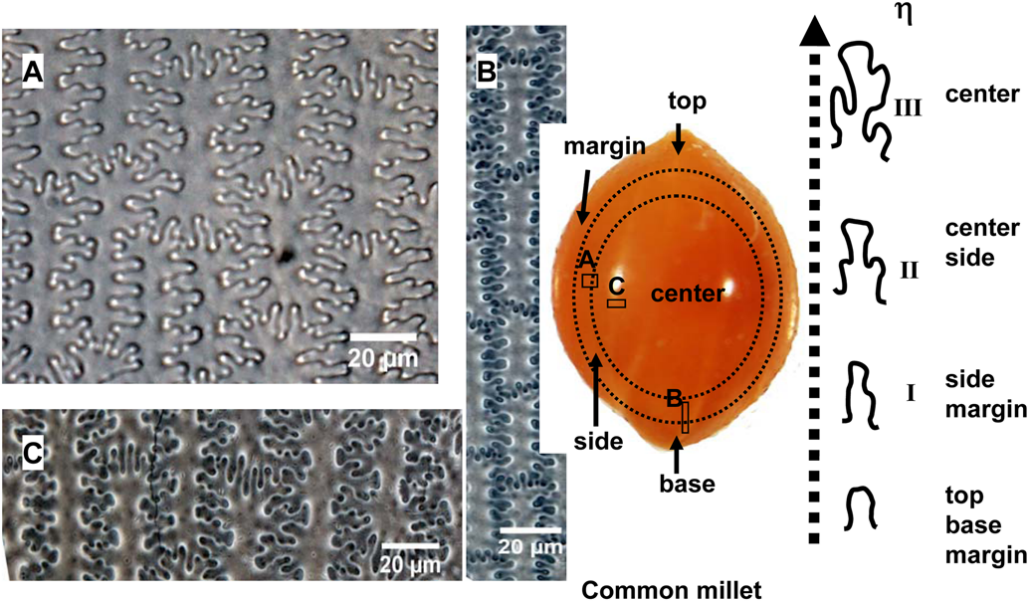
Undulated patterns transformation of epidermal long cell walls in the upper lemma and palea of Common millet. (A), (B), and (C) showing the different designs of phytoliths at side, base to side, and center of lemma of Common millet, respectively.

The undulations tend to increase in highly sinuous variation toward the central part of the lemmas and palea, where the undulations of the long cell walls produce branching subordinate Ω (Ω II, III) or η (ηII, III) sinuous margins that join the margins across the cells. The different Ω-undulated regular patterns occur at different parts by gradual change in a general way from base and top (Ω I), to side (ΩII), to center (Ω III) of the lemmas and palea in Foxtail millet (Figure 7). Similarly, the different η-undulated patterns also occur at different parts of the lemmas and palea of Common millet, by gradual change from base and top (η I), to side (ηII), to center (η III) (Figure 8).

This suggests that the surface undulated patterns of epidermal long cell walls in the upper lemma and palea can be used to distinguish between Foxtail millet and Common millet. The epidermal long cell walls are Ω-undulated (Ω-I, II, III) in *S. italica*, and η-undulated (η-I, II, III) in *P. miliaceum*.

#### The endings structures of epidermal long cell in the upper lemma and palea

**Figure 9:**
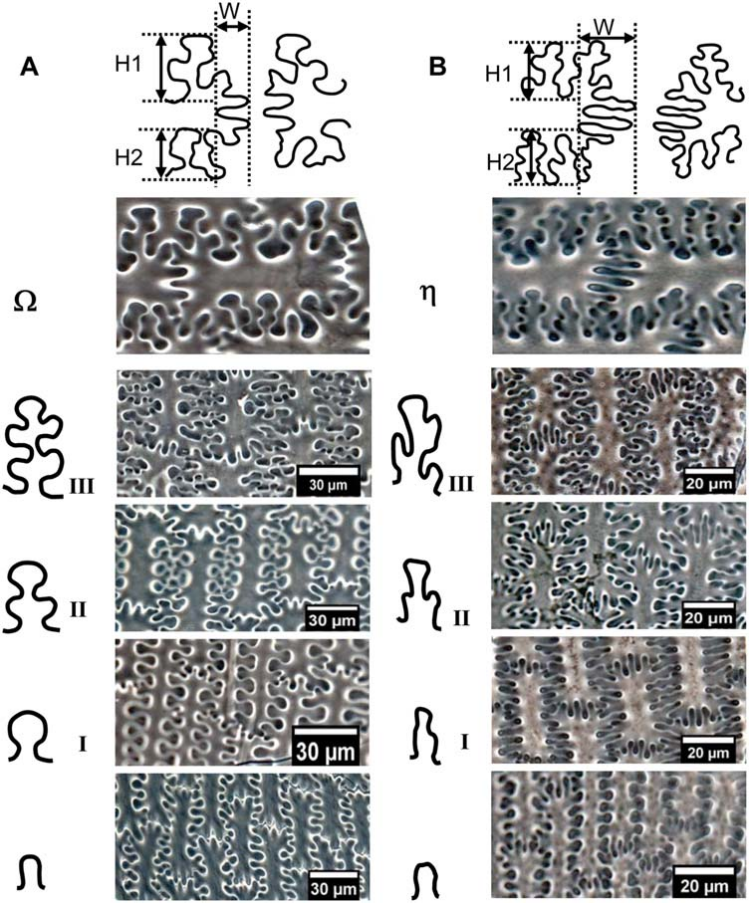
Comparison of the endings structures of epidermal long cells in the upper lemma and palea for the two millet species. (A) Cross wavy type of Foxtail millet. (B) Cross finger type of Common millet.

Based on our observation and statistics of endings structures of epidermal long cells, we found that three important parameters can be used to characterize the morphological variations of structures of epidermal long cells in the upper lemma and palea (Figure 9): (1) W = width of endings interdigitation of dendriform epidermal long cells. (2) H = undulation amplitude of dendriform epidermal long cell walls. (3) R = ratio of width of endings interdigitation to undulation amplitude, R = W/((H1+H2)/2) (Figure 9). These three parameters are relatively stable among different millet samples.

**Figure 10:**
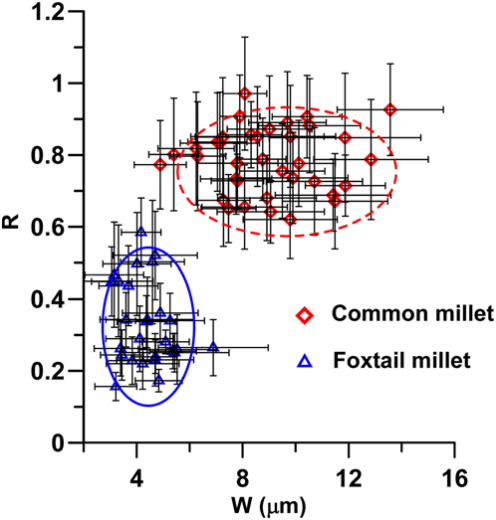
Bivariate biplot of R and W values of measurements from epidermal long cells of both species (*P. miliaceum* and *S. italica*).

We divide endings structures of epidermal long cell into Cross wavy type and Cross finger type based on characteristics of the dendriform epidermal long cell endings joining others (Figure 9). Cross wavy type, dendriform epidermal long cell endings joining others in a wavy pattern, is formed in the upper lemma and palea of *S. italica*. The average width of endings interdigitation of dendriform epidermal long cells is about 4.37±0.89 µm (N = 2774) (Figure 10) (Table 2). Cross finger type, dendriform epidermal long cell endings joining others in a deeply digital pattern, is formed in the upper lemma and palea of *P. miliaceum*. However, the average width of endings interdigitation of dendriform epidermal long cells is longer (8.95±2.02 µm, N = 3303) in the Cross finger type of *P. miliaceum* than that in *S. italica*. (Figure 10).

**Table 2:** Measured data of dendriform epidermal long cells for modern Common millet and Foxtail millet.

Plant samples	W (µm)		(H1+H2)/2 (µm)		R		Count Number
	Average	SD	Average	SD	Average	SD	N.
P 1-1*	5.40	0.94	6.86	1.21	0.80	0.16	103
P 1-2	7.76	1.35	10.59	1.30	0.73	0.10	106
P 1-3	8.92	1.86	13.15	1.88	0.68	0.12	110
P 2-1	7.47	1.04	11.58	1.42	0.65	0.09	84
P 2-2	8.07	1.61	12.38	1.50	0.66	0.12	176
P 2-3	9.79	1.77	15.83	1.40	0.62	0.11	99
P 3-1	7.14	1.49	8.63	1.51	0.83	0.14	152
P 3-2	9.50	1.61	12.68	1.60	0.76	0.13	135
P 3-3	11.88	1.51	16.64	1.17	0.71	0.09	120
P 4-1	7.89	0.76	8.77	0.92	0.91	0.11	77
P 4-2	9.69	1.46	10.96	1.19	0.89	0.14	69
P 4-3	8.75	0.88	11.18	0.69	0.79	0.11	59
P 5-1	7.25	1.04	8.65	0.94	0.85	0.17	71
P 5-2	12.85	2.16	16.55	1.79	0.79	0.17	114
P 5-3	11.41	2.18	16.55	1.53	0.69	0.10	67
P 6-1	4.89	0.98	7.29	0.75	0.77	0.12	69
P 6-2	7.80	0.99	10.80	1.56	0.73	0.07	67
P 6-3	10.71	1.82	14.95	1.96	0.73	0.15	87
P 7-1	8.56	2.51	10.12	2.77	0.85	0.14	97
P 7-2	9.90	2.08	13.47	1.89	0.74	0.13	97
P 7-3	11.48	1.99	17.21	1.03	0.67	0.13	80
P 8-1	6.24	1.44	7.62	0.89	0.82	0.16	107
P 8-2	8.32	1.16	9.72	0.86	0.86	0.09	80
P 8-3	7.28	1.00	10.93	0.97	0.67	0.13	92
P 9-1	7.05	1.12	8.54	1.39	0.84	0.14	76
P 9-2	9.80	1.71	11.53	1.04	0.85	0.14	84
P 9-3	9.06	2.02	14.13	2.70	0.64	0.09	69
P 10-1	6.30	1.06	8.09	1.62	0.80	0.15	90
P 10-2	7.79	1.54	10.05	1.12	0.78	0.13	91
P 10-3	10.12	1.38	13.15	1.56	0.78	0.12	75
P 11-1	9.01	1.60	10.41	1.40	0.87	0.15	86
P 11-2	10.53	1.92	11.92	1.13	0.88	0.13	93
P 11-3	11.86	2.86	14.01	1.71	0.85	0.18	88
P 12-1	8.08	0.82	8.45	1.07	0.97	0.16	82
P 12-2	10.43	1.32	11.56	1.17	0.91	0.11	77
P 12-3	13.57	2.01	14.72	1.60	0.93	0.13	74
S1-1**	4.01	1.29	8.12	1.56	0.50	0.14	83
S 1-2	4.89	1.43	13.54	2.45	0.36	0.08	95
S 1-3	5.53	1.47	22.25	4.11	0.26	0.10	103
S 2-1	4.60	1.21	7.75	1.06	0.50	0.17	71
S 2-2	3.45	0.81	15.17	4.42	0.24	0.07	95
S 2-3	3.22	0.79	20.64	2.02	0.16	0.04	94
S 3-1	4.17	0.75	7.15	0.69	0.58	0.10	72
S 3-2	4.37	1.11	12.91	1.50	0.34	0.07	87
S 3-3	4.23	1.38	19.42	2.68	0.22	0.07	83
S 4-1	3.16	1.11	6.86	1.36	0.47	0.15	69
S 4-2	3.40	1.00	12.97	1.06	0.26	0.08	71
S 4-3	5.37	0.89	21.18	1.15	0.26	0.05	66
S 5-1	4.70	1.59	9.04	2.12	0.52	0.12	104
S 5-2	5.26	1.30	15.68	3.14	0.34	0.08	103
S 5-3	4.70	1.46	20.70	3.44	0.23	0.07	113
S 6-1	3.06	0.76	6.86	1.27	0.45	0.10	82
S 6-2	5.09	0.89	28.29	2.31	0.28	0.03	66
S 6-3	3.84	0.96	16.96	1.09	0.23	0.07	88
S 7-1	3.31	1.30	7.43	1.79	0.45	0.15	200
S 7-2	4.12	1.28	14.46	2.61	0.29	0.09	219
S 7-3	5.43	2.05	21.38	5.49	0.25	0.05	125
S 8-1	3.69	1.11	8.53	1.80	0.44	0.11	152
S 8-2	4.40	1.78	14.09	6.56	0.34	0.12	152
S 8-3	6.89	2.07	26.24	4.44	0.26	0.08	134
S 9-1	3.60	0.76	10.67	1.67	0.34	0.08	104
S 9-2	4.71	0.80	19.80	1.45	0.24	0.05	67
S 9-3	4.84	0.88	28.05	2.09	0.17	0.03	76

(W = width of endings interdigitation of dendriform epidermal long cells. H = undulations amplitude of dendriform epidermal long cell walls. R = ratios of width of endings interdigitation to undulations amplitude).

Notes:

* Px-y: P = *Panicum miliaceum* L.; Px (x = 1–12) corresponding to No. in Table 1; y = 1, 2, 3 correspond to ηI, ηII, and ηIII types (see Figure. 6), respectively.

** Sx-z; S = *Setaria italica* (L.) Beauv.; Sx (x = 1–9) corresponding to No. in Table 1; z = 1, 2, 3 correspond to ΩI, ΩII, and ΩIII types (see Figure. 6), respectively.

Figure 10 shows the bivariate biplot by s-coordinates of the 3303 measurements from epidermal long cells of *P. miliaceum* and 2774 measurements from those of *S. italica*, plotted along axis W and axis R, and their classification into two groups corresponding to the two species (*P. miliaceum* and *S. italica*). The R-value is higher (0.79±0.12, N = 3303) in *P. miliaceum* than in *S. italica* (0.33±0.11, N = 2774) (Figure 10) (Table 2).

#### The surface sculpture of epidermal long cells in the upper lemma

Diverse silicon deposits can occur at different cell layers, including extracellular sheet (keratose layer), outer epidermis, hypoderm fibres, vascular bundle, and occasional silicification of internal spongy mesophyll in the transection of lemma and palea [23].

**Figure 11:**
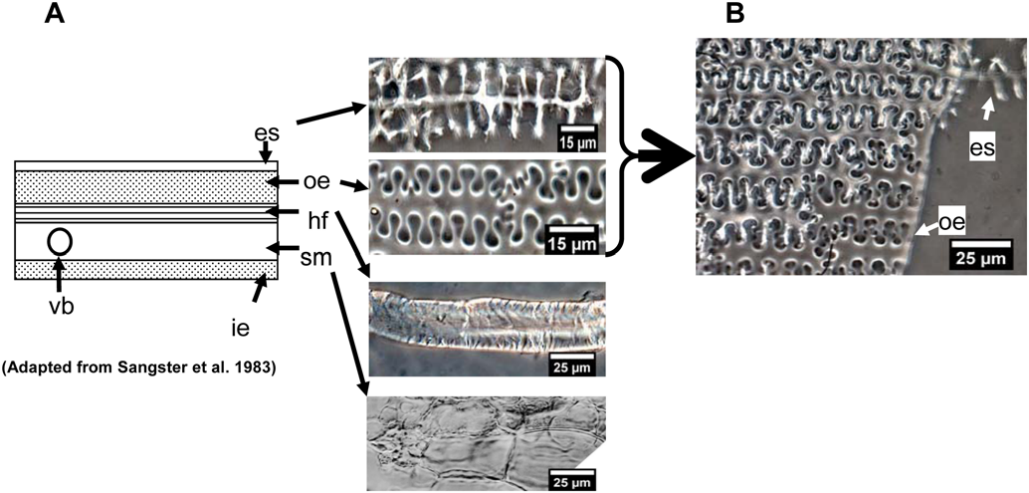
Diverse silicon deposits occur at different cell layers in epidermal long cell of the upper lemma from Foxtail millet. (A) Transection of lemma and palea of *S. italica* showing the following: es: extracellular sheet (keratose layer), oe: outer epidermis, hf: hypoderm fibres, vb: vascular bundle, sm: spongy mesophyll, and ie: inner epidermis. (B) Heavy silicon surface ridgy line sculpture with adnate silicon extracellular sheet and outer epidermis.

Surface ridgy line sculpture of the upper lemma is important for the identification of *S. italica*, which is characterized by having an adnate silicon extracellular sheet and outer epidermis, forming a very heavy silicon layer (Figure 11).

**Figure 12:**
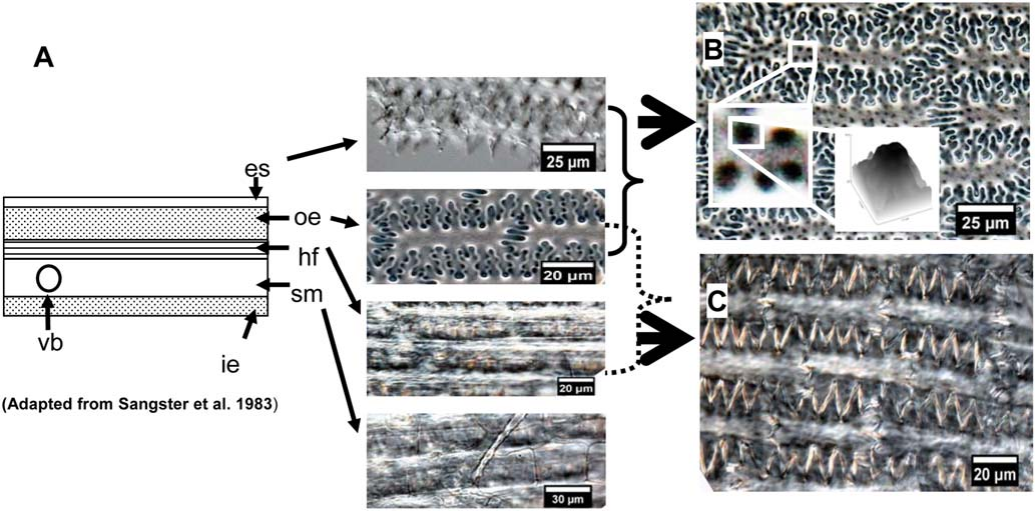
Diverse silicon deposits occur at different cell layers in epidermal long cell of the upper lemma from Common millet. (A) Transection of lemma and palea of *P. miliaceum* showing the following: es: extracellular sheet (keratose layer), oe: outer epidermis, hf: hypoderm fibres, vb: vascular bundle, sm: spongy mesophyll, and ie: inner epidermis. (B) Surface spotted sculpture with adnate silicon extracellular sheet and outer epidermis. (C) Surface saw-toothed sculpture with adnate silicon outer epidermis and hypoderm fibres.

*P. miliaceum* have a smooth spotted sculpture with adnate silicon extracellular sheet and outer epidermis, or a surface saw-toothed sculpture with adnate silicon outer epidermis and hypoderm fibres. This is a reliable feature in distinguishing them from *S. italica* (Figure 12).

**Figure 13:**
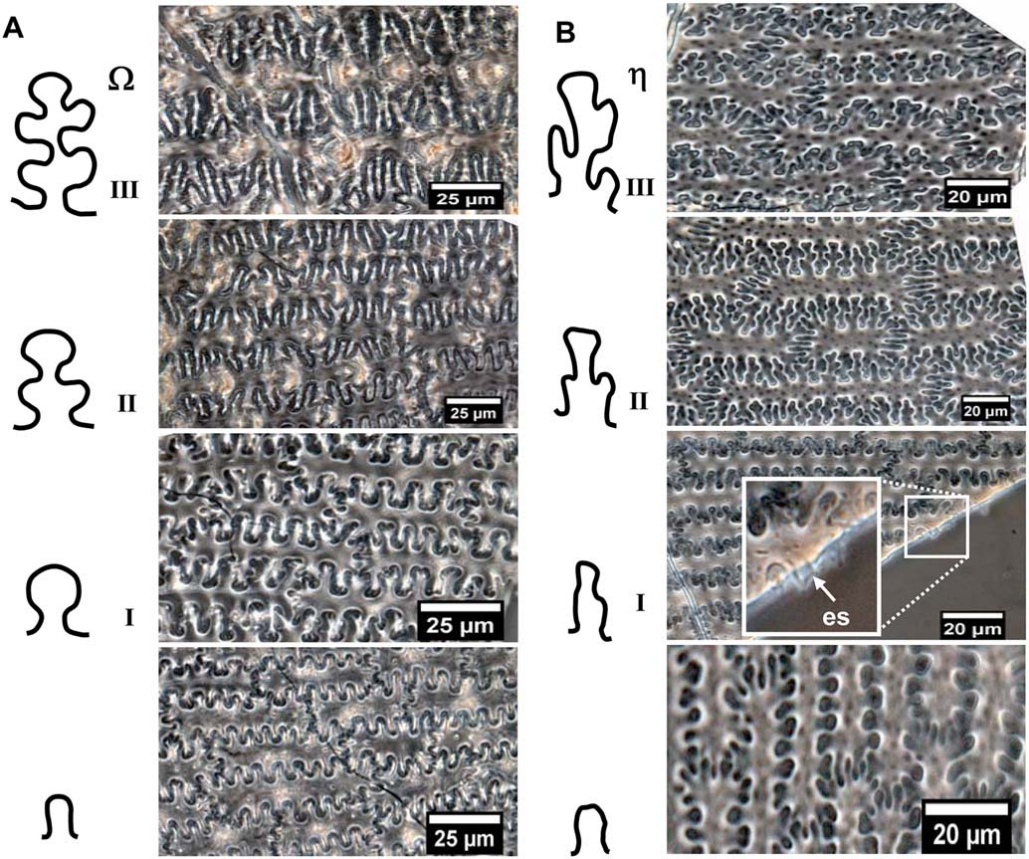
Comparison of the adnate silicon surface sculpture in the upper lemma for two millets. (A) Heavy silicon surface ridgy line sculpture in different Ω-types from Foxtail millet. (B) Surface spotted sculpture in different η-types from Common millet. es: extracellular sheet (keratose layer).

Based on our observation of surface characteristic with different adnate silicon layers in different Ω-types or η-types, we found that the surface ridgy line sculpture of the upper lemma is peculiar to *S. italica* (Figure 13).

### Preliminary contrast of phytolith morphology between millets and related grasses

**Figure 14:**
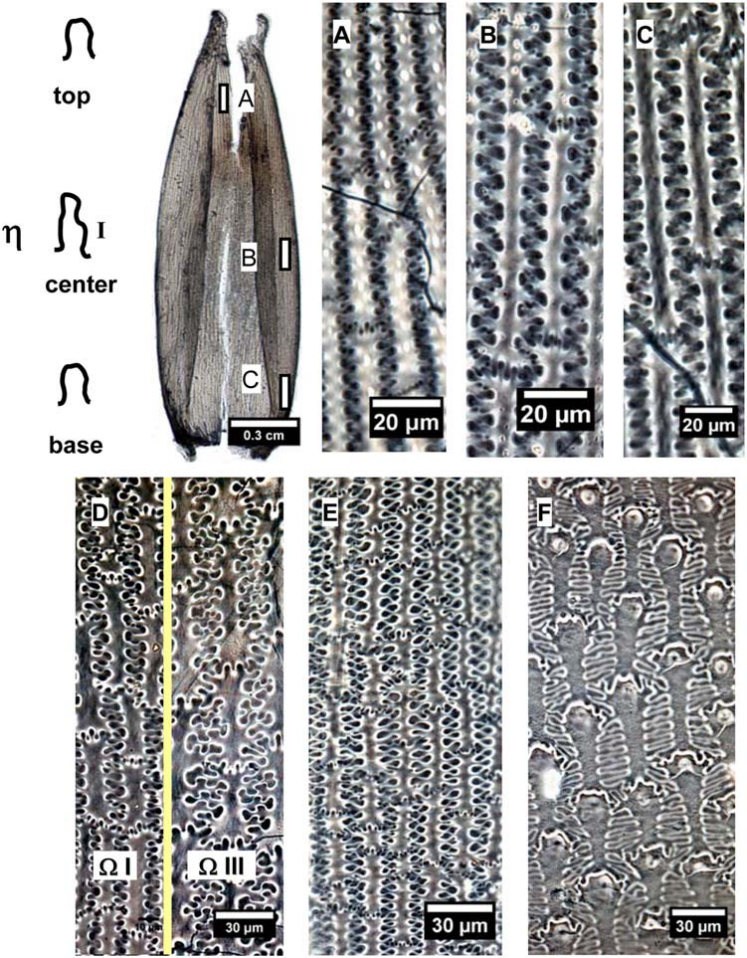
Comparison of micromorphology of lemma for *P. bisulcatum* (A), (B), (C), *S. italica* (D), *S. viridis* (E), and *S. plicata* (F).

The phylogenetic relationship of Eurasian *Panicum* species is currently unknown, and the wild ancestor of *P. miliaceum*, if it still exists, has not been conclusively identified. *Panicum bisulcatum* Thunb., a species of wild grass in China potentially related to *P. miliaceum*, can be distinguished from *P. miliaceum* based on its phytolith characteristics, because it typically has simple obvious silica skeleton (ηI type) (Figure 14A, B, C) that is distinct from the well-defined ηII-III type in *P. miliaceum*.

The wild ancestor of Foxtail millet (*S. italica*) is presumed to have originated from *S. viridis* (green foxtail), a ubiquitous weed from the Eurasian continent [17]. We examined the silicon structure patterns in the glumes, lemmas, and paleas from the inflorescence bracts in modern Foxtail millet, and closely related grasses, including *S. viridis*, *S. plicata* (Lam.) T. Cooke. Figure 14 shows that foldaway ∞-undulated pattern occurs in *S. viridis* (Figure 14E) and the multiped worm sculpture pattern occurs in *S. plicata* (Figure 14F). A species-specific identification of phytoliths is possible for *S. italica* (Figure 14D) and *P. miliaceum* because they have typically well-defined silica skeletons that are distinguishable from those in *P. bisulcatum*, *S. viridis*, and *S. plicata*, which have no such demonstrable patterns, additional studies are needed to confirm the observations.

## Discussion

Early investigators have reported the potential of using grain shapes (expressed as the length-to-breadth ratio, and morphological variations) for discriminating between *P. miliaceum* and *S. italica* [19], and between wild and domesticated *S. italica* [30]–[32]. However, the grain shape analysis is ineffective in discriminating between Foxtail millet and Common millet, because their grains are very small in size compared to wheat or barley, and their chaff is more delicate and similar to each other. Moreover, the overlapping ranges of the length-to-breadth ratios between *S. italica* and *P. miliaceum* make the identification of at least charred de-husked grains of the domesticated species difficult [8], [20], [21]. Other studies also reported on the variation of bilobes/crosses phytolith within leaves from *Panicum* sp. and *Setaria* sp. [33]–[35], however, the morphological characteristics of bilobes/crosses are not sufficient to distinguish *S. italica* from *P. miliaceum*.

The inflorescence bracts are well known sites of heavy silicification in cereals, due to the hot and arid habitat of the cereals, conditions that promote intensive transpiration and water loss and lead to the close formation of phytoliths in inflorescence bract cell [2]. Most of the silicon in the inflorescence bracts has been concentrated in the outer (abaxial) epidermis, but the precise location of the heaviest deposits varies with the species. As mentioned above, early investigators have recognized the potential of using inflorescence phytoliths for discriminating between wheat and barley [10], [28], wild and domesticated *Oryza* [6], [12], *Avena sativa* and *A. strigosa* [36], and many other grasses [23], [37]–[39].

Foxtail millet and Common millet are vitally important food crops for people living in the Far East and even in the entire Eurasian continent prior to the popularity of rice and wheat [15], [40], [41]. However, the previous studies show that there is no valid method for separating Foxtail millet and Common millet based on inflorescence phytolith analysis [8], [20], [22]. These are several reasons for rendering the previous identifications questionable. One of the main obstacles in inflorescence phytolith systematics is that perplexing variations in morphology occur from tissue to tissue in spikelet, and from apex to base within lemma or glume, and in specific tissues. Another reason is that diverse silicon deposits can occur at different cell layers in the transection of lemma and palea. The complication surface sculptures of upper lemmas with adnate different silicon layers have not been discussed in detail. As a result of very little work conducted on the phytoliths, no clear diagnostic feature has been found and used to distinguish between Foxtail millet and Common millet.

Because of this, we had to dissect the spikelet of modern plants into five parts, including lower glume, upper glume, lower lemma (lemma of sterile floret), upper lemma, and palea, to examine the variation of anatomy and silicon structure patterns in different parts, from base to center to apex, and margins, cover with whole glumes, lemmas, and paleas. Our observation and statistics of the micromorphology throughout each glume, lemma, and palea reveal well-regulated variations in phytoliths between Foxtail millet and common millet, particularly regarding the presence or absence of papillae, undulated patterns of epidermal long cell, and surface ridgy line sculpture. Based on a large sample, the average width of endings interdigitation of dendriform epidermal long cells examined in a large number is found to be consistently and significantly different between the two species examined and therefore can be considered a diagnostic feature to distinguish between *S. italica* and *P. miliaceum*. This character is as significant in the taxonomy of each genus as other quantitatively important characters.

By all accounts, our research indicates that five key diagnostic characteristics in phytolith morphology could be used to distinguish Foxtail millet from Common millet (Table 3): (i) Cross-shaped type is formed in the lower lemma and glumes of *S. italica*, whereas Bilobe-shaped type is formed in those of *P. miliaceum*. (ii) Regularly arranged papillae on the surface of the upper lemma and palea are peculiar to *S. italica*. (iii) The epidermal long cell walls are Ω-undulated (Ω-I, II, III) in *S. italica*, and η-undulated (η-I, II, III) in *P. miliaceum*. (iv) Cross wavy type (dendriform epidermal long cell endings joining others in a wavy pattern) occurs in the upper lemma and palea of *S. italica*, whereas Cross finger type (dendriform epidermal long cell endings joining others in a deeply digital pattern) is formed in those of *P. miliaceum*. The R value (ratio of the width of endings interdigitation to the amplitude of undulations) is higher (0.79±0.12, N = 3303) in *P. miliaceum* than in *S. italica* (0.33±0.11, N = 2774). (v) Surface ridgy line sculpture of the upper lemma is also important for the identification of *S. italica*, which is characterized by having an adnate silicon extracellular sheet and outer epidermis, forming a very heavy silicon layer that is a reliable feature in distinguishing them from *P. miliaceum*. These five diagnostic characteristics used together give the only reliable way of distinguishing Foxtail millet from Common millet. A species-specific identification of phytoliths is possible for *S. italica* and *P. miliaceum* because they have typically well-defined silica skeletons that are distinguishable from those in *P. bisulcatum*, *S. viridis*, and *S. plicata*, which have no such demonstrable patterns.

**Table 3:** Comparison of the characteristics of phytoliths in inflorescences bracts for the Foxtail millet and Common millet.

Parts of Spikelet		Foxtail millet	Common millet
**Lower lemma and glumes**	The shape of silica bodies	Cross-shaped type	Bilobe-shaped type
**Upper Lemma and palea**	The presence or absence of papillae	Regularly arranged papillae	Smooth surface without any papillae
	The undulated patterns of epidermal long cells	Ω-undulated (Ω-I, II, III)	η-undulated (η-I, II, III)
	The endings structures of epidermal long cells	Cross wavy type	Cross finger type
		W = 4.37±0.89 µm	W = 8.95±2.02 µm
		R = 0.33±0.11	R = 0.79±0.12
	The surface sculpture	Surface ridgy line sculpture	Smooth spotted sculpture or saw-toothed sculpture

The results of this study have revealed distinct differences between Foxtail millet and Common millet. Nevertheless, several caveats exist in the morphological characteristics of phytoliths, which should be mentioned. A number of factors may influence the within-individual variations in phytolith morphology, including the stage of plant maturity [42], intraspecific variation within the plant taxa [1], [2], [13], [43], the amount of soluble silica in local ground water [33], the rate of tissue transpiration [44], the tissue within which the phytoliths form [43], [45], the location of phytoliths in leaf blades [43], [46], genetic variation among plants, and geographic location where the plants grew [43], [47]. Additional phytolith studies, particularly those that concentrate on variation within a single species in different seasons and regions, will help provide more efficient and more accurate methods.

It is not the focus of this paper to discuss in detail how phytoliths can be used to distinguish millets from other related grasses. Although the phytolith production patterns revealed in our preliminary research give encouraging results that may point the way to distinguishing the millets from related grasses in China, more research is needed, especially the study of more wild species and landraces of domesticated millet species. To be of practical use to investigators, further morphometric analysis of a wide variety of millet species is required. Future work could use this methodology to develop classification paradigms, and to gain an understanding of the effect of domestication and polyploidization on phytolith morphometries.

### Conclusions

In discussions of the origin of early agriculture, Foxtail millet and Common millet have received particular attention, since they were the dominant traditional crops in the Far East and even in the entire Eurasia. However, until now, the identification and taxonomic distinction between Foxtail millet and Common millet in archaeobotanical remains had been problematic, especially because the crop grains preserve only when charred, or where their preservation is poor.

This is the first study of the variation of the anatomy and silicon structure patterns in the glumes, lemmas, and paleas occurring among 27 modern millets and related grass species collected from different regions in China. We found that five key diagnostic characteristics in phytolith morphology could be used to distinguish between Foxtail millet and Common millet, as follows. (i) Cross-shaped and Bilobe-shaped are formed in *S. italica* and *P. miliaceum*, respectively; (ii) Papillae on the upper lemma and pales are peculiar to *S. italica*; (iii) The epidermal long cell walls are Ω-undulated in *S. italica*, and η-undulated in *P. miliaceum*; (iv) The endings structures of epidermal long cells are Cross wavy type in *S. italica*, and Cross finger type in *P. miliaceum*; (v) Surface ridgy line sculpture of the upper lemma are peculiar to *S. italica*. Collectively, these five diagnostic characteristics provide the only reliable way of distinguishing Foxtail millet from Common millet. A species-specific identification of phytolith is possible for *S. italica* and *P. miliaceum* because they have typically well-defined silica skeletons that are distinguishable from those in *P. bisulcatum*, *S. viridis*, and *S. plicata*, which have no such demonstrable patterns.

This practical protocols, if supported by additional studies of phytoliths derived from more millets and related grass species, can be give the only reliable way of separating the Common millet, Foxtail millet, and other related grass species based on their phytoliths.

## References

[1] Piperno DR (1988). Phytolith analysis: An archaeological and geological perspective.

[2] Piperno DR (2006). Phytoliths: A Comprehensive Guide for Archaeologists and Paleoecologists.

[3] Pearsall DM (2000). Paleoethnobotany: A handbook of procedures (2nd ed.).

[4] Lu HY, Liu KB (2003). Morphological variations of lobate phytoliths from grasses in China and the southeastern U.S.A.. Divers Distrib.

[5] Lu HY, Yang XY, Ye ML, Liu KB, Xia ZK (2005). Millet noodles in Late Neolithic China.. Nature.

[6] Zhao ZJ, Pearsall DM, Benfer RA, Piperno DR (1998). Distinguishing rice (*Oryza sativa* Poaceae) from wild Oryza species through phytolith analysis II: finalised method.. Econ Bot.

[7] Ball TB, Gardner JS, Brotherson JD (1996). Identifying phytoliths produced by the inflorescence bracts of three species of wheat (*Triticum monococcum* L., *T. dicoccum* Schrank., and *T. aestivum* L.) using computer-assisted image and statistical analyses.. J Archaeol Sci.

[8] Harvey EL, Fuller DQ (2005). Investigating crop processing using phytolith analysis: the example of rice and millets.. J Archaeol Sci.

[9] Madella M, Weber S, Belcher WR (2003). Investigating agriculture and environment in South Asia: present and future contributions from opal phytoliths.. Indus Ethnobiology: New Perspectives from the Field.

[10] Rosen AM, Weiner S (1994). Identifying ancient irrigation: a new method using opaline phytoliths from emmer wheat.. J Archaeol Sci.

[11] Itzstein-Davey F, Taylor D, Dodson J, Atahan P, Zheng HB (2007). Wild and domesticated forms of rice (*Oryza* sp.) in early agriculture at Qingpu, lower Yangtze, China: evidence from phytoliths.. J Archaeol Sci.

[12] Pearsall DM, Piperno DR, Dinan EH, Umlauf M, Zhao Z (1995). Distinguishing rice (*Oryza sativa* Poaceae) from wild Oryza species through phytolith analysis: results of preliminary research.. Econ Bot.

[13] Piperno DR (1984). A comparison and differentiation of phytoliths from maize and wild grasses: use of morphological criteria.. American Antiquity.

[14] You XL (1993). The question for origin and spread in both Foxtail millet and Common millet.. Agriculture History of China.

[15] Bellwood P (2005). First Farmers: The Origins of Agricultural Societies.

[16] Zheng DS (2005). Use of cereal crop wild relatives in crop breeding in China.. J Plant Genetic Resources.

[17] Lu TLD (1998). Some botanical characteristics of green foxtail (*Setaria viridis*) and harvesting experiments on the grass.. Antiquity.

[18] Crawford GW, Stark MT (2006). East Asian Plant Domestication.. Archaeology of Asia.

[19] Liu CJ, Kong ZC (2004). Morphological comparison of Foxtail millet and brookcorn millet and its significance in archaeological identification.. Kaogu [Archaeology].

[20] Zhao ZJ (2006). Domestication of millet–paleoethnobotanic data and ecological perspective.. Archaeology in China and Sweden.

[21] Fuller DQ (2006). Agricultural Origins and Frontiers in South Asia: A Working Synthesis.. J World Prehist.

[22] Parry DW, Hodson MJ (1982). Silica distribution in the caryopsis and inflorescences bracts of Foxtail millet (*Setaria italica* (L.) Beauv.) and its possible significance in carcinogenesis.. Ann Bot.

[23] Sangster AG, Hodson MJ, Wynn Parry D (1983). Silicon deposition and anatomical studies in the inflorescence bracts of four *Phalaris* species with their possible relevance to carcinogenesis.. New Phytol.

[24] Wynn Pahrry D, Smithson' F (1966). Opaline silka in the inflorescences of some British grasses and cereals.. Ann Bot.

[25] Terrell EE, Wergin WP (1981). Epidermal features and siiica deposition in lemmas and awns of *Zizania* (Gramineae).. Am J Bot.

[26] Wynn Parry D, Hodson MJ (1982). Silica distribution in the caryopsis and inflorescence bracts of Foxtail millet (*Setaria italica* (L.) Beauv.) and its possible signiflcance in carcinogenesis.. Ann Bot.

[27] Tubb HJ, Hodson MJ, Hodson GC (1993). The inflorescence papillae of the Triticeae: A new tool for taxonomic and archaeological research.. Ann Bot.

[28] Ball TB, Gardner JS, Anderson N (1999). Identifying inflorescence phytoliths from selected species of wheat (*Triticum monococcum*, *T. dicoccon*, *T. dicoccoides*, and *T. aestivum*) and barley (*Hordeum vulgare* and *H. spontaneum*) (Gramineae).. Am J Bot.

[29] Berlin AM, Ball T, Thompson R, Kittleson D, Herbert SC (2003). Ptolemaic agriculture, “Syrian wheat”, and *Triticum aestivum*.. J Archaeol Sci.

[30] Nasu H, Momohara A, Yasuda Y, He JJ (2007). The occurrence and identification of *Setaria italica* (L.) P. Beauv. (Foxtail millet) grains from the Chengtoushan site (ca. 5800 cal B.P.) in central China, with reference to the domestication centre in Asia.. Veget Hist Archaeobot.

[31] Musil AF (1963). Identification of crop and weed seeds (Agriculture Handbook 219).

[32] Renfrew JM (1973). Palaeoethnobotany: the prehistoric food plants of the Near East and Europe.

[33] Wang YJ, Lu HY (1993). The study of phytolith and its application.

[34] Lu HY (1998). Quaternary environmental changes recorded by magnetic susceptibility and plant fossils: quantitative estimates of paleoclimates..

[35] Krishnan S, Samson NP, Ravichandran P, Narasimhan D, Dayanandan P (2000). Phytoliths of Indian grasses and their potential use in identification.. Bot J Linn Soc.

[36] Portillo M, Ball T, Manwaring J (2006). Morphometric analysis of inflorescence phytoliths produced by *Avena sativa* L. and *Avena strigosa* Schreb.. Econ Bot.

[37] Acedo C, Liamas F (2001). Variation of micromophological characters of lemma and palea in the genus *Bromus* (Poaceae).. Ann Bot Fennici.

[38] Jacobs SWL (2001). The genus Lachnagrostis (Gramineae) in Australia.. Telopea.

[39] Costea M, Tardif FJ (2002). Taxonomy of the most common weedy European *Echinochloa* species (Poaceae: Panicoideae) with special emphasis on characters of the lemma and caryopsis.. Sida.

[40] Crawford GW, Cowan CW, Watson PJ (1992). Prehistoric plant domestication in East Asia.. The Origins of Agriculture.

[41] Nesbitt M, Prance G (2005). Grains.. Cultural History of Plants.

[42] Hodson MG, Sangster AG, Parry DW (1985). An ultrastructural study on the developmental phases and silicification of the glumes of *Phalaris canariensis* L.. Ann Bot.

[43] Mulholland SC, Rapp JRG, Olledorf AL (1988). Variation in phytoliths from corn leaves.. Can J Bot.

[44] Whang SS, Kim K, Hess WM (1998). Variation of silica bodies in leaf epidermal long cells within and among seventeen species of *Oryza* (Poaceae).. Am J Bot.

[45] Ball TB, Brotherson JD, Gardner JS (1993). A typologic and morphometric study of variation in phytoliths from einkorn wheat (*Triticum monococcum*).. Can J Bot.

[46] Takeoka Y, Wada T, Naito K, Kaufman PB (1984). Studies on silicification of epidermal tissues of grasses as investigated by soft X-ray image analysis. II. Differences in frequency of silica bodies in bulliform cells at different positions in the leaves of rice plants.. Jap J Crop Sci.

[47] Mulholland SC, Rapp JRG, Regal R (1990). Variation in phytoliths within a population of corn (Mandan Yellow Flour).. Can J of Bot.

